# Eukaryotic-like phosphoprotein phosphatase (PPP) enzyme evolution: interactions with environmental toxins and regulatory proteins

**DOI:** 10.1042/BSR20230378

**Published:** 2023-05-23

**Authors:** David Kerk, Chris White-Gloria, Jayde J. Johnson, Greg B. Moorhead

**Affiliations:** Department of Biological Sciences, University of Calgary, Calgary, T2N 1N4, Canada

**Keywords:** evolutionary biology, okadaic acid, protein phosphatases

## Abstract

Phosphoprotein phosphatases (PPPs) are a ubiquitous class of enzymes which dephosphorylate serine and threonine residues on substrate proteins involved in a wide variety of cellular processes. The active site of PPP enzymes are highly conserved with key residues coordinating the substrate phosphoryl group (the two R-clamp) and two metal ions necessary for catalysis. Because of the diverse number of roles that these enzymes play it is no surprise that they are highly regulated in the cell, often accomplished by binding regulatory subunits. These regulatory subunits are able to dictate substrate specificity, localization, and activity of the bound catalytic subunit. Eukaryotic PPP subtypes have been previously shown to manifest varying degrees of sensitivity to environmental toxins. We present here an evolutionary model which now rationalizes this data. Our re-examination of published structural evidence reveals that Eukaryotic PPP toxin-binding residues also interact with substrate binding residues (the two R-clamp) and ancient regulatory proteins. Such functional interactions could have stabilized PPP sequence early in Eukaryotic evolution, providing a stable target which was co-opted by toxins and their producer organisms.

## Introduction

Eukaryotes employ reversible protein phosphorylation to control a variety of cellular processes. Protein phosphorylation can regulate protein localization, protein–protein interactions, catalytic activity, and protein turnover [1]. In this light, a conservative estimate suggests that over 70% of Eukaryotic proteins are phosphorylated at some point in their lifespan [2]. Indeed, a significant portion, 2–4%, of the eukaryotic genome is dedicated to coding the enzymes in charge of protein phosphorylation. Protein kinases catalyze the addition of a phosphoryl group to serine and threonine (96–98%) and to a lesser extent, tyrosine residues (2–4%) [2,3]. Protein phosphatases work antagonistically to these kinases by catalyzing the hydrolysis of the phosphate moiety. While once thought to play a passive ‘housekeeping’ role in managing protein phosphorylation status in the cell [4], over the past few decades protein phosphatases have increasingly been the subject of numerous studies linking them to regulatory aspects of cell function and growth [5,6].

Protein phosphatases are classified into four sequence distinct families believed to have all evolved independently in Eukaryotes [7–9]. These include the phosphoprotein phosphatases (PPP), the phosphoprotein Mn^2+^/Mg^2+^-dependent metallophosphatases (PPM), the protein tyrosine phosphatases, and the aspartate-based protein phosphatases [9]. The most well studied family of protein phosphatases is the PPP family which includes protein phosphatase 1 (PP1), as well as PP2A, PP2B/calcineurin/PP3, PP4, PP5, PP6, PP7 and a group we refer to as PP7-like [5,10]. In Eukaryotic cells, it is these proteins which account for ∼90% of all protein dephosphorylation on serine/threonine residues [4,11]. It is understood that PPPs associate with regulatory subunits and when compared to the PPMs, PPP inhibitors such as okadaic acid and microcystin-LR are generally much more potent to this class of enzyme [12].

In previous work we traced a likely evolutionary history of PPP enzymes from a phosphodiesterase ancestor to a monoesterase specific for phospho-proteins [13]. Subsequently, we uncovered Eukaryotic-like PPP enzymes in bacteria and archaea and demonstrated a likely archaeal ancestor in the Asgard superphylum [10]. All these Eukaryotic-like PPP enzymes have extremely well conserved active site residues and architecture that coordinate two metal ions for catalysis and cradle the substrate phosphoryl-oxygens with two arginine residues (2-Arg clamp) [10,13]. This ‘locked in’ architecture and set of catalytic residues poses a problem for substrate specificity that undoubtedly contributed to evolution of PPP regulatory subunits that confer substrate specificity and a means to regulate activity [4,10]. Multiple highly conserved subunits are known for PP1, PP2A, PP3, PP4 and PP6 [4,5].

To date, well over 200 regulatory subunits have been discovered to dock PP1 in humans with many of them being conserved across Eukaryotes, and others being specific to certain groups of organisms (e.g., yeast or mammals) [4,5,9,14,15]. For PP1, the most common regulatory subunit anchoring site is the RVxF SLiM with other/additional binding sites known (G/SILK, ΦΦ and AF) [16]. In addition, these regulatory subunits also often make use of the acidic, hydrophobic and C-terminal grooves on PP1, which are found near its active site [4]. Today, knowledge of PPP active sites, regulatory subunit binding regions and motifs are often exploited for drug design to inhibit protein dephosphorylation [17–21]. It is therefore no surprise that evolution has given rise to biological toxins with an analogous function.

Relatively early in the context of evolution, certain bacteria and unicellular eukaryotes acquired the ability to produce toxins which target members of the PPP family. These toxins can be classified as polyketide-based linear or peptide-based cyclic molecules. Okadaic acid, tautomycin and tautomycetin are structurally similar linear toxins with unique hydrophobic ends containing either a spiroketal or alkene functional group. The differences in the hydrophobic ends largely account for these toxins' binding affinity to PPP members [22,23]. These toxins inhibit their targets through active site and hydrophobic grove binding. Peptides microcystin and calyculin A bind the same area on PPPs utilizing some of the same PPP residues used for docking the aforementioned toxins [23–25]. Through a structurally and bioinformatically founded literature search, we determined ten PPP residues crucial to toxin binding. With this in hand, we consider the origin of the diverse sensitivity of Eukaryotic PPPs to these environmental toxins and rationalize these data with an evolutionary model. Finally, we highlight the role of Eukaryotic PPP toxin-binding residues in PPP interactions with critical modulator proteins and present a hypothesis for the origin of PPP sequence conservation underlying toxin effects.

## Methodology

### PPP candidate sequences and multiple sequence alignment

**Figure 1:**
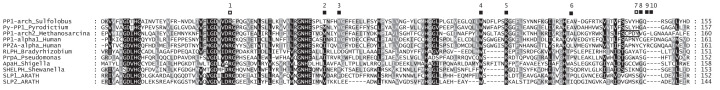
Toxin sensitivity residues in PPP sequences An alignment was made using 300 sequences, including bacterial PPPs (phosphoprotein phosphatases), Eukaryotic PPPs, and ‘Eukaryotic-Like’ PPPs from both Bacteria and Archaea as described in Methods. Sequences were ultimately culled (see Methods) to reduce this alignment to the representative sequences indicated in this Figure. The accession numbers for the remaining sequences are: ‘PP1-alpha_Human’ (1FJM_A), ‘PP2A-alpha_Human’ (2IAE_C), ‘RLPH_Bradyrhizobium’ (H5YEZ7), ‘PrpA_Pseudomonas’ (NP_745164.1), ‘ApaH_Shigella’ (2DFJ_A), ‘SHELPH_Shewanella’ (A0KU52). Residues marked by filled squares are important for binding of both MC-LR and OA. Residues marked with open squares have been shown to bind MC-LR, OA, and the PP1 protein inhibitor I-2. The boxed region before and including Site 7 of the *Methanosarcina* sequence highlights its similarity to that of the Eukaryotic PP1 and PP2A sequences.

The MAFFT server [26], https://mafft.cbrc.jp/alignment/software/) was used to generate candidate multiple sequence alignments. These were produced under a variety of alignment options and evaluated both quantitatively (using the TCS function of the T-Coffee server ([27], http://tcoffee.crg.cat/apps/tcoffee/index.html)) and qualitatively). An alignment was made using MAFFT (BLOSUM45, EINSI options) of 300 sequences, including bacterial PPPs (phosphoprotein phosphatases), Eukaryotic PPPs and ‘Eukaryotic-Like’ PPPs from both Bacteria and Archaea as described in [10,13]. The MAFFT-Add feature (BLOSUM45, LINSI) was used to add to this alignment ‘SLP1_ARATH’ (Q8L774) and ‘SLP2_ARATH’ (Q944L7). Taxa were then culled to reduce this alignment to one including the three archaeal PPPs whose toxin-sensitivity has been studied biochemically (‘PP1-arch_Sulfolobus’ [Q55059], ‘Py-PP1_Pyrodictium’ (O08367), ‘PP1-arch2_Methanosarcina’ (O34200)), ten representative PP1s (cd07414), ten representative PP2As (cd07415), ten representative bacterial PrpA_PrpBs (cd07424), ten representative bacterial ApaHs (cd07422), seven bacterial RLPHs, and four bacterial SHELPHs. This alignment was then edited by hand to remove poorly aligned regions. The TCS score of this edited precursor alignment was 847. Taxa were then culled again to reduce this alignment to the sequences indicated in Figure 1. Homologues of PP1 and PP2A regulatory proteins (Supplemental Table S1) were obtained using HHBlits [28].

### Phylogenetic tree inference

A rooted tree was inferred with BEAST (Bayesian Evolutionary Analysis by Sampling Trees) [29] v. 1.8.3, run at the CIPRES Science Gateway ([30], http://www.phylo.org/). BEAUTi v. 1.8.2 was used to prepare controlling XML files locally. The evolutionary model LG+G8 was used (LG+G16 is not available), along with a log-normal relaxed uncorrelated clock [31]. MCMC (Markov Chain Monte Carlo) was generally run for 200–250m cycles. Data were combined from the runs of two independent chains, with the first 10% (1000) of each tree set (10,000 total) manually excluded as burn-in.

### Candidate sequences to map eukaryotic PPP toxin-sensitivity

For assessing the distribution and characteristics of Eukaryotic PPP subtypes, a composite protein database from 45 representative Eukaryotes was assembled as described in [10]. A representative PP1, PP2A, PP4, PP6 and PP5 was found for each of the 45 species. The phylogenetic range of Bsu1, PP7, PP2B and PPEF/RdgC was not universal and in addition we uncovered a novel PPP subtype we designated PP7-like [10]. Sequences were aligned as described above and used to generate a rooted Bayesian tree.

### Identification of candidate PPP toxin-binding residues

Representative PPP sequences gathered as above were aligned with MAFFT, BLOSUM45 and EINSI. PP1 and PP2A residues binding toxins (particularly okadaic acid (OA)) were obtained from the literature [32,33] and used to identify corresponding aligned residues in other sequences.

### Identification of PPP toxin-binding residues involved in interactions with Modulator Proteins

The RCSB PDB (Protein Data Bank) ([34], https://www.rcsb.org/) was searched for all solved structures containing PP1 or PP2A and known regulatory subunits or proteins that modify the PP2A catalytic subunit. The coordinate files for each solved structure were downloaded, and protein interactions visualized using PyMOL. Attention was focused on the set of OA-binding residues identified as described above, and the residues of the conserved β12-β13 loop region. Localization of toxin-binding residues within described regulator motifs was achieved through inspection of the literature, and alignment of additional sequences as necessary using MAFFT, BLOSUM45, LINSI.

### Structural analysis and modeling using AlphaFold

Structures were visualized using the PyMOL Molecular Graphics System, Version 1.2r3pre, Schrödinger, LLC. AtPP7 and PP7-like protein structure was modelled using AlphaFold [35].

## Results

### Evolution of PPP sensitivity to environmental toxins

**Figure 2:**
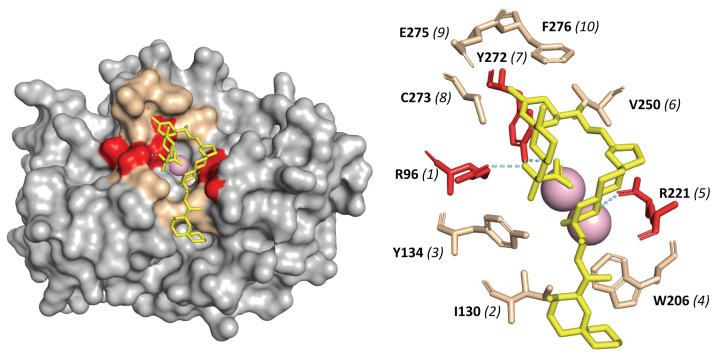
Human PP1γ interacts with okadaic acid (OA) using 10 conserved toxin binding sites Surface representation of PP1γ showing the active site defined by metal ions (purple spheres) and bound okadaic acid (yellow). Key okadaic acid binding residues are indicated with red and beige (left). On the right, okadaic acid and the 10 key toxin binding residues (sites 1-10) in PP1 are labelled having polar (red) and non-polar (beige) interactions with OA. Sites 1-10 from Figure 1 are indicated with brackets, residues labelled by single amino acid code and numbered according to human PP1γ. Hydrogen-bonding between sites (1), (5) and (7) and OA hydroxyl groups shown with blue dashed lines. Interaction (PDB: 1JK7) visualized in PyMOL.

**Figure 3:**
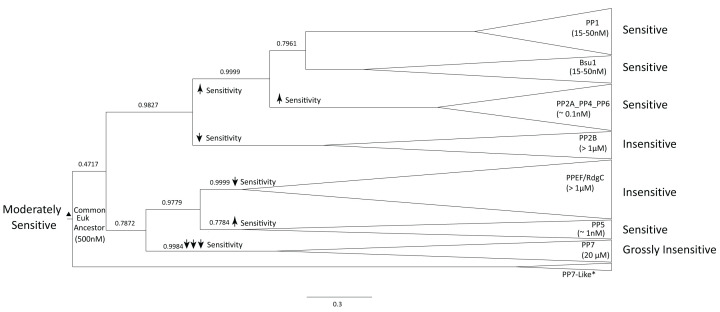
Evolution of toxin-sensitivity in Eukaryotic PPP sequences Reference Eukaryotic PPP (phosphoprotein phosphatase) sequences as described in [10] were used to search a database from a panel of 45 completely sequenced Eukaryotic genomes utilizing Eukaryotic PPP HMMs (Hidden Markov Models). Sequences for Eukaryotic PPP subtypes were collected from an iterative database search utilizing Eukaryotic PPP HMMs [10]. Sequences were aligned, and a rooted Bayesian tree inferred as detailed in [10]. For clarity of presentation, sequence groups were collapsed into cartoon wedges. Support values are posterior probabilities. Eukaryotic PPP subtypes are indicated. ‘PP7-Like’ sequences are discussed in the text. Okadaic acid sensitivities (IC50s) are given for each sequence group. The triangle symbol indicates the Eukaryotic common ancestor PPP, which is assumed to have the same okadaic acid sensitivity as the most sensitive known archaeal PPP sequence (PP1-arch2 of *Methanosarcina*). Up or down arrows indicate an increase or decrease in okadaic acid sensitivity, respectively. Okadaic acid sensitivity * of PP7-like is predicted (see main text). A detailed Toxin-Sensitivity Evolution Tree for Eukaryotic PPPs is presented in Supplementary Figure S2.

Several types of Eukaryotic PPPs have been shown to be exquisitely sensitive to environmental toxins, perhaps the best studied examples being microcystin (produced primarily by the cyanobacterium *Microcystic aeruginosa*) and okadaic acid (produced by dinoflagellates of the genera *Procentrum* and *Dinophysis*) [25,36]. A variety of studies, utilizing both directed mutagenesis of expressed sequences and solved protein-toxin structures, have identified phosphatase residues critical to toxin binding and sensitivity. There have been more limited studies of the effects of these toxins on prokaryotic PPPs. Bacterial PPPs are reported to be insensitive (IC50 > 1 micromolar), whereas the few archaeal PPPs which have been studied appear to have intermediate sensitivity. We sought to investigate the possible evolution of PPP toxin sensitivity by mapping known toxin-binding residues onto a sequence alignment containing selected, but representative, Eukaryotic, bacterial, and archaeal PPP sequences. The results are summarized in Figures 1 and 2, as well as Table 1. We have focused our literature summary on the phosphatases PP1 and PP2A, and the toxin okadaic acid. The archaeal PPP phosphatase sequences we have aligned are those which have been studied for toxin sensitivity *in vitro*. Their names (‘PP1-arch’, ‘Py-PP1’ and ‘PP1-arch2’) reflect their nomenclature in the relevant literature [37–39]. It should be noted that each of these archaeal sequences is of the ‘Eukaryotic-like PPP’ type [10], as analyzed in Figure 1. Sequence ‘PP1-arch2_Methanosarcina’ has been shown to have the greatest toxin sensitivity of any archaeal sequence studied thus far and is identical to our sequence O34200. For Eukaryotic PPP–OA interactions, ten sites have been identified as important. Examination of the distribution of aligned residues within bacterial and archaeal sequences reveals a very interesting pattern. Sites 1 (R96 of PP1α [human PP1α]), 2 (I130 of PP1α), 4 (W206 of PP1α), and 5 (R221 of PP1α) are nearly universally conserved amongst the aligned sequences. This suggests that these residues may be considered as ‘permissive’ for toxin binding (i.e. necessary but not sufficient). Notably, R96 and R221 of PP1α are the key arginine residues that cradle the two substrate phosphoryl group oxygens and are nearly universally conserved in PPP enzymes [10]. Site 3 (Y134 of PP1α) is shared between eukaryotes and all the archaeal sequences studied. Mutation at this site might thus be considered ‘enabling’ – contributing to the intermediate toxin sensitivity previously noted in archaeal PPP proteins. Site 7 (Y272 of PP1α) is shared only between the Eukaryotic PPPs and ‘PP1-arch2_Methanosarcina’. This might be considered another ‘enabling’ mutation. Site 10 (F276 of PP1α) corresponds to an L in ‘PP1-arch2_Methanosarcina’, which is the only archaeal PPP which has a residue aligned at this position. This could possibly be another ‘enabling’ mutation in this sequence. Site 6 (V250 of PP1α), site 8 (C273 of PP1α) and site 9 (E275 of PP1α) are not shared by any of the archaeal sequences. Hence, these positions might be considered important for the greatly enhanced sensitivity of Eukaryotic PPPs toward okadaic acid. Sites 1-10 of PP1α and their interaction with okadaic acid were visualized in PyMOL and presented in Figure 2 illustrating the role and location of each of the 10 sites in human PP1α. Finally, inspection of the alignment in Figure 1 shows the marked similarity in the boxed region preceding and including Site 7 of the *Methanosarcina* sequence (PP1-arch2) to that of the Eukaryotic PP1 and PP2A sequences. This strongly supports the tree topology presented in Figures 2 and 3 of our previous work [10], where the ‘Eukaryotic-Like PPPs’ of the *Methanosarcinales* group are most closely related to Eukaryotic PPPs.

**Table 1:** Summary of Toxin Sensitivity Residues in PPP Sequences

Sequence	Site 1	Site 2	Site 3	Site 4	Site 5	Site 6	Site 7	Site 8	Site 9	Site 10
PP1-arch_Sulfolobus	✓	-	✓	✓	✓	-	-	-	-	-
Py-PP1_Pyrodictium	✓	✓	✓	✓	✓	-	-	-	-	-
PP1-arch2_Methanosarcina	✓	✓	✓	✓	✓	-	✓	-	-	✓?
PP1-alpha_Human	✓	✓	✓	✓	✓	✓	✓	✓	✓	✓
PP2A-alpha_Human	✓	✓	✓	✓	✓	✓	✓	✓	✓	✓
RLPH_Bradyrhizobium	✓	✓	-	✓	✓	-	-	-	-	-
PrpA_Pseudomonas	✓	✓	-	✓	✓	-	-	-	-	-
ApaH_Shigella	✓	✓	-	✓	✓	-	-	-	-	-
SHELPH_Shewanella	✓	✓	✓	✓	✓	-	-	-	-	-
SLP1_ARATH	✓	✓	-	✓	✓	-	-	-	-	-
SLP2_ARATH	✓	✓	-	✓	✓	✓	-	-	-	-

A sequence alignment was constructed as given in the legend to Figure 1. This table summarizes the presence or absence of residues at key toxin-binding sites cited in the literature.

Sites 6-10 are discussed in the text. Literature citations are given in the text.

### Evolution of toxin-binding diversity within eukaryotic PPPs

Our data summarizing the alteration of environmental toxin-binding residues in the origin of Eukaryotic PPPs, and our data expanding the phylogenetic distribution of Eukaryotic PPP types [10], then led us to examine toxin-binding in the context of the Eukaryotic PPP radiation. First, we aligned the sequence of PP1-arch2 from *Methanosarcina thermophila* with those of all Eukaryotic PPPs whose sensitivity to okadaic acid has been directly characterized. We focused our attention on the set of 10 toxin-binding residues highlighted in our above analysis. The results are presented as Table 2. Inspection of this data shows that of the ten positions, residues at Site 6, and Sites 8, 9, and 10 are most important to toxin sensitivity, especially the latter three positions. Each position is characterized by amino acid residue types which yield higher toxin sensitivity: for Site 6 – C or hydrophobic; for Site 8, C; for Site 9, polar or charged; for Site 10, C or hydrophobic. Residues in the table that are red conflict with these tendencies and are therefore associated with reduced toxin sensitivity. Thus, the Archaeal sequence has an unfavorable residue at Site 8, and a deletion at Site 9. PP2B/PP3 has unfavorable residues at all three of Sites 8, 9, and 10. PPEF has unfavorable residues at Site 8 and Site 10. PP7 has unfavorable residues at Sites 6, 8 and 10. In addition, PP7 has insertions at two places in the polypeptide between Sites 5 and 6, and between Sites 7 and 8. Although no solved structure of PP7 is available, it might be expected that these insertions would alter the geometry of the toxin-binding regions of the protein. We explored this possibility by modelling PP7 using AlphaFold [35], overlaying the structure with human PP1γ and visualizing the residue side chains equivalent to sites 1-10 (Supplementary Figure S1). In addition to the primary sequence violations of sites 6, 8, and 10 (Table 2), modelled PP7 overlayed on PP1γ reveals the structural misalignment of sites 8 and 9. Inserts in the PP7 sequence, not present in other PPP members, cause the spatial position of these residues to be shifted away from toxin binding. Hence, it is not surprising that PP7 is the most toxin-insensitive of all the Eukaryotic PPP types. An alignment and model of two PP7-like enzymes [10] suggests that like PP7, they would be grossly insensitive to okadaic acid (data not shown). In terms of Table 2, it appears that PP1 and PP2A should be approximately equivalent in their okadaic acid sensitivity. Yet, PP2A is more than 100× more sensitive. This has been investigated previously and has been attributed to the enhanced ‘hydrophobic cage’ with which the PP2A molecule interacts with the hydrophobic end of okadaic acid [32].

**Table 2:** Evolution of Toxin-Binding Diversity Within Eukaryotic PPPs

Phosphatase	OA IC_50_	OA Sens	Site 1	Site 2	Site 3	Site 4	Site 5	Site 6	Site 7	Site 8	Site 9	Site 10
PP1-arch2	500 nM	Mod Sens	R	M	Y	W	R	**A**	Y	**V**	-	**L**
PP1	15–50 nM	Sens	R	I	Y	W	R	**V**	Y	**C**	**E**	**F**
BSU1	15–50 nM	Sens	R	M	Y	W	R	**C**	Y	**C**	**T**	**A**
PP2A	0.1 nM	Sens	R	I	Y	W	R	**L**	Y	**C**	**R**	**C**
PP2B	>1 μM	Insens	R	L	F	W	R	**A**	Y	**L**	**V**	**Y**
PP7	20 μM	Gross Insens	R	C	Y	W	R	**G** ^1^	Y	**E** ^2^	**R**	**Y**
PP5	∼1 nM	Sens	R	M	Y	W	R	**V**	Y	**C**	**Q**	**M**
PPEF1	>1 μM	Insens	R	M	Y	W	R	**C**	Y	**Y**	**E**	**G**
Consensus for sensitive sites						C or Φ best		C best	Polar or charged best	C or Φ best		

PPP sequences were retrieved from the UniProt database and aligned using MAFFT, with BLOSUM45 and EINSI option. Aligned residues were retrieved for the ten important toxin-binding positions identified previously and are shown as single letter amino acid codes. Sites which are **bold** (sites **6**, **8**, **9** and **10**) indicate the most critical positions for evolved differences in toxin-sensitivity. The sequence accession numbers were: PP1-arch2 (O34200); PP1γ (P36873); BSU1 (Q9LR78); PP2Aα (P67775); PP2Bα (Q08209); PP7 (Q9FN02); PP5 (P53041); PPEF1 (O14829). Abbreviations used: ‘Seq’ = Sequence; ‘OA’ = Okadaic acid; ‘OA Sens’ = Okadaic acid sensitivity; ‘Mod Sens’ = Moderately sensitive; ‘Sens’ = Sensitive; ‘Insens’ = Insensitive; ‘Gross Insens’ = Grossly insensitive; ‘**–’** = Deletion at this position; ‘Φ’ = Hydrophobic residue. Residues in RED indicate violations of consensus pattern at that aligned position for a ‘Sensitive’ response.

1 PP7 has an insert of 12 residues before this site.

2 PP7 has an insertion of 6 residues before this site.

We constructed a rooted Bayesian tree showing the direction of evolution of the various Eukaryotic PPP subclasses and mapped onto it the known IC50 values for okadaic acid. The result is shown in Figure 3 (a more detailed version of this tree is presented as Supplementary Figure S2). We have assumed a sensitivity of the ancestral Eukaryotic PPP to be similar to that of the closest known Archaeal sequence, that of PP1-arch2 from *Methanosarcina thermophila*, which we have termed ‘moderately sensitive’. We have also assumed a parsimonious pattern of descent, where toxin sensitivity would be inherited from the Eukaryotic PPP common ancestor and modified to reach various interior tree branch points. Given these starting assumptions, and the shape of the tree, it is plausible to envision that the sorts of alterations to the evolving Eukaryotic PPP subtypes listed in Table 2 would lead to the observed pattern of toxin sensitivity.

### Eukaryotic PPPs use toxin-binding residues to interact with protein modulators

Having traced a plausible route for the establishment of the observed spectrum of okadaic acid sensitivities in evolving Eukaryotic PPP subtypes through alteration in a set of toxin-binding residues, we sought to explore possible normal functions of these PPP residues. We reasoned as follows. First, our phylogenetic distribution data [10], indicates that both PP2A and PP1 originated early in Eukaryotic evolution. Second, each of these proteins displays a high degree of sequence conservation between Eukaryotes. This suggests functional constraints on an early ancestral sequence of each type, usually attributed to a fixed active site architecture and to interactions with multiple protein modulators. When combined with their high toxin sensitivity, this suggests the possibility that toxin binding might utilize residues previously selected to perform vital functional roles.

To explore this hypothesis, we examined the role of the ten toxin-binding residues previously catalogued for PP2A and PP1 in their interactions with protein modulators and/or regulatory subunits. We also examined other residues in the conserved β12-β13 loop which have been extensively studied for toxin interactions. We visualized the interactions in the solved co-structures of PP2A or PP1 with other proteins using PyMOL. Since we were looking at the possibility of toxin-binding residues performing other binding functions, we restricted our analyses to toxin-free structures. Our results are summarized in Supplemental Table 1. We found a remarkable number of interactions occurring at the residues of interest between PP2A and its (de)methylating enzymes: LCMT-1 (structure 3P71) (10 interactions) and PME-1 (structure 3C5W) (7 interactions). We found a range of interactions between PP1 and several of its regulators, with I-2 (structure 2O8A) having the most (8 interactions), followed by PNUTS (structure 4MOY) (7 interactions), and 6 interactions each for Spinophilin (structure 3EGG) and Neurabin-1 (structure 3HVQ). Regulator R15B had 5 interactions (structure 4V0X), or 3 interactions (structures 4V0U and 4V0V). Finally, Ki67 had two interactions (structure 5J28) and RepoMan had a single interaction (structures 5INB and 5IOH). Amongst the PP1 regulators, I-2 had interactions restricted to between residues 141 and 152 of its 206 amino acid length. In contrast, the other PP1 regulators had interactions within or very close to previously characterized binding motifs: the ‘Arg’ motif of PNUTS, Spinophilin, Neurabin-1 and R15B, and the ‘KiR-SLiM’ of Ki67 and RepoMan (Supplementary Table S1).

At least one interaction was identified with 9 of the 10 toxin-binding sites (the exception being no interaction with Site 2). At least one interaction was identified at 8 of the 13 residues in the β12-β13 loop (note that Sites 7–10 are also in this loop). Of the 10 toxin-binding sites, the most frequently used were Site 1 and Site 3 (used by all regulators except Ki67 and RepoMan). Additional β12-β13 loop residues used most frequently were: P270, used by all PP1 regulators except I-2; and N271 used by the same subset of PP1 regulators with the equivalent residues being used by the PP2A regulator LCMT-1. It should be stressed that these interactions represent a subset, not the entirety of the PPP-modulator protein interactions observed in each solved co-structure.

Finally, we worked to obtain a quick proxy metric for the likely evolutionary age of each of the interacting proteins in this dataset. We performed HHBlits searches with the modulator/regulatory protein and characterized the phylogenetic distribution of likely homologues (hits encompassing the PPP interacting region, with high probability of being a true positive [*P*>94%]). This is also shown in Supplementary Table S1. Both PP2A interacting proteins (LCMT-1 and PME-1) and the PP1 interacting protein I-2 have a very diverse and widespread homologue distribution, suggesting they arose very early in Eukaryotic evolution, likely at the Eukaryotic origin. The distribution of PNUTS is less extensive, indicating an ancient origin but perhaps not at the origin of Eukaryotes. In contrast, all the rest of the PP1 regulatory proteins are restricted to Metazoa, implying a somewhat more recent evolutionary origin.

## Discussion

### The origin and diversification of environmental toxin sensitivity in eukaryotic PPPs

The extraordinary sensitivity of many Eukaryotic PPPs to various environmental toxins has been known for some time. However, the evolutionary origin of this sensitivity, and indeed the role of such toxins within their native ecosystems, has remained elusive. Characterization of three of the archaeal PPPs has allowed us to trace the origin of toxin sensitivity. Our data suggest that Eukaryotic PPP toxin sensitivity was acquired stepwise, progressing from binding residues shared by all PPPs, through those shared by Eukaryotic and archaeal sequences only, to a final set unique to Eukaryotes alone. Inspection of Figure 2, where PP1 toxin-binding residues are mapped in their association with okadaic acid, yields a fascinating observation. What we have called Site 10 (F276 in PP1) forms hydrophobic contacts with the OA molecule at substituents off its C10 and C13. This is a site where the most sensitive archaeal PPP (‘PP1-arch2’ from *Methanosarcina thermophila*) also has a hydrophobic residue (L). We classified Site 6 (V250 of PP1), Site 8 (C273 of PP1) and Site 9 (E275 of PP1) as the final set of Eukaryotic-specific mutations which produce high Eukaryotic PPP sensitivity to OA. It is remarkable that in the solved PP1–OA structure, these three residues also form hydrophobic interactions with precisely the same portion of the OA molecule as does Site 10. Hence it is conceivable that these mutations might serve to increase OA binding affinity and hence increase toxin sensitivity in addition to the interaction already established by the residue at Site 10. What this suggests is that the OA molecule itself need not have changed structure in order for it to have differential effects on Eukaryotic, as opposed to archaeal, PPPs. Thus, it is conceivable that OA might have evolved, at least in part, to inhibit archaea in the ecosystem of the producer dinoflagellates. In this view, the enhanced sensitivity of PPPs of Eukaryotes would then be an unfortunate consequence of the acquisition of further mutations (presumably selected for organismal-specific functional reasons) also stabilizing OA binding.

We further traced the evolution of toxin sensitivity through the several varieties of Eukaryotic PPPs. When combined with a rooted Bayesian phylogeny, we derived a plausible scheme whereby the various levels of toxin sensitivity or insensitivity could be satisfactorily reconciled. It is important to emphasize that a key feature of this model is the assumption that the earliest Eukaryotic PPP common ancestor had a toxin sensitivity similar to that of the most sensitive characterized Archaeal sequence, that from PP1-arch2 of *Methanosarcina thermophila* (a member of the group of Archaeal PPPs most closely related to Eukaryotic PPPs as shown in Figures 2 and 3 of [10]. This provides a starting point which can be readily modified by modest sequence changes toward either higher or lower toxin sensitivity. This is important to account, for example, for the PP5/PPEF divergence, with resulting greatly differing toxin sensitivities.

### Eukaryotic PPP protein modulation and toxin binding

Eukaryotic PPPs differ greatly in their molecular organization and modes of regulation. The polypeptide chains of PP5s, PPEFs, and Bsu1s possess accessory domains (TPR, EF-hand, and Kelch-like, respectively), which have been shown to be involved in regulation of catalytic activity and substrate specificity of their attached phosphatase domains. In contrast, PP1, PP2A, and PP2B operate through holoenzyme complexes where a catalytic subunit is modulated by suites of interacting subunits. In particular, PP1 is known to interact with over 200 regulatory proteins. This often occurs through binding of a conserved ‘RVxF’ motif to a hydrophobic cleft on PP1. In addition, other regulators have been shown to interact with various PP1 surface features (G/SILK, ΦΦ and AF), the combination of which has been referred to as the ‘PP1-binding code’ [11]. It is speculated that many more PP1 regulatory subunits still await discovery, and it is likely that additional binding motifs will emerge [16].

The data we have harvested from published protein structures demonstrate that a subset of residues which PP1 and PP2A use to interact with functionally essential modulator proteins have been previously characterized as toxin-binding residues. We found that several well-studied toxin binding sites were involved in the interaction of PP2A with (de)methylating enzymes methyltransferase LCMT-1 [40] and methylesterase PME-1 [41]. Methylation of PP2A catalytic subunit (as well the subfamily members PP4 and PP6) carboxyl-terminal leucine controls holoenzyme complex assembly [42,43]. Similarly, we found that such toxin-binding sites on PP1 interacted with I-2, an important ancient regulator which inhibits the enzyme by blocking substrates from the active site [44]. We also found toxin-binding site interaction with several regulators which confer PP1 substrate specificity by modulating substrate access to the C-terminal binding groove: PNUTS (‘PP1 Nuclear Targeting Subunit’), Spinophilin and Neurabin-1, Ki67 and RepoMan. The likely ancient origin of PP1 regulators strongly suggests that the toxins, and their producer organisms, co-opted functionally conserved target residues for their own purposes.

**Figure 4:**
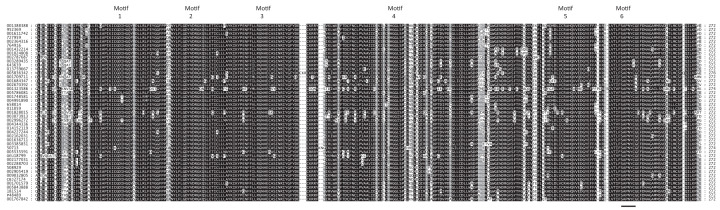
Alignment of Eukaryotic PP1 Sequences A reference set of PP1 sequences from 45 representative Eukaryotic species was collected as detailed in Methods. A MAFFT alignment with BLOSUM45 and the EINSI option was produced and is depicted here. Conserved motifs are indicated (see Figure 1 of (10)). The ‘SAPNYC’ region described in the text is underlined in motif 6.

The interactions manifested by regulatory proteins with previously characterized toxin-binding residues of PP1 may help explain one of its signature sequence features, the ‘SAPNYC’ motif. This is part of the β12-β13 loop, the full sequence comprising 13 residues. An examination of a sequence alignment of PP1s from 45 phylogenetically diverse Eukaryotes (Figure 4) demonstrates that this region is very nearly invariant. This suggests some strong functional constraint, probably established very early in Eukaryotic evolution. The interaction of PP1 with regulatory proteins would be a logical candidate for such a mechanism.

### Hypotheses and future directions

Based on the observations presented here and elsewhere, we hypothesize that PPP family enzymes had an established active site architecture early in Eukaryotic evolution. Up to 10 residues are utilized by okadaic acid to bind and potently inhibit PP2A family members, PP1 and BSU1. Other PPP enzymes, which display reduced toxin inhibition, have fewer than 10 and/or less optimal toxin binding amino acids at these positions. To achieve substrate specificity, PPP regulatory subunits or modifying proteins exploit these same conserved sites to bind catalytic subunits. This is particularly notable in the ancient PP1 inhibitor I-2 and the PP2A methylating and demethylating enzymes that utilize 7 to 10 of these key toxin-binding sites. Having a fixed active site and adopting the use of regulatory/modifying proteins early in Eukaryotic evolution, it is likely that toxin-producing organisms exploited this stable target.

We comprehensively investigated okadaic acid due to the number of enzyme inhibitor studies it has been used in and the structural biology work that examines its docking site on PP1 and PP2A. Future work should focus on expressing additional archaeal PPP enzymes, determining their sensitivity to okadaic acid and other toxins, and relating this back to the observations made here. In addition, more structures of PPP enzymes with other toxins would aid in confirming the ideas presented in this article. To initiate our study, we focused on okadaic acid but acknowledge that complexes of PP1 with several other toxins are available in the PDB and make an obvious next step in supporting the results presented here.

## Supplementary Material

Supplementary Figures S1-S2

Supplementary Table S1

## Data Availability

Sequence groups and individual sequences are available as supplementary files in (10, 13) and data will be made available by the corresponding author on reasonable request.

## Abbreviations

At: Arabidopsis thaliana
IC50: inhibitor concentration producing 50% inhibition of enzyme activity
MC-LR: microcystin-LR
OA: okadaic acid
PPP: phosphoprotein phosphatase
SLiM: Short Linear Motif
