# Supplementary material for: Eukaryotic-like phosphoprotein phosphatase (PPP) enzyme evolution: interactions with environmental toxins and regulatory proteins
Source: Biosci Rep. 2023 May 23;43(5):BSR20230378. doi: 10.1042/BSR20230378 (PMC10214084; doi:10.1042/BSR20230378)
Supplement: Supplementary Figures S1-S2 [file BSR-2023-0378_supp.pdf]

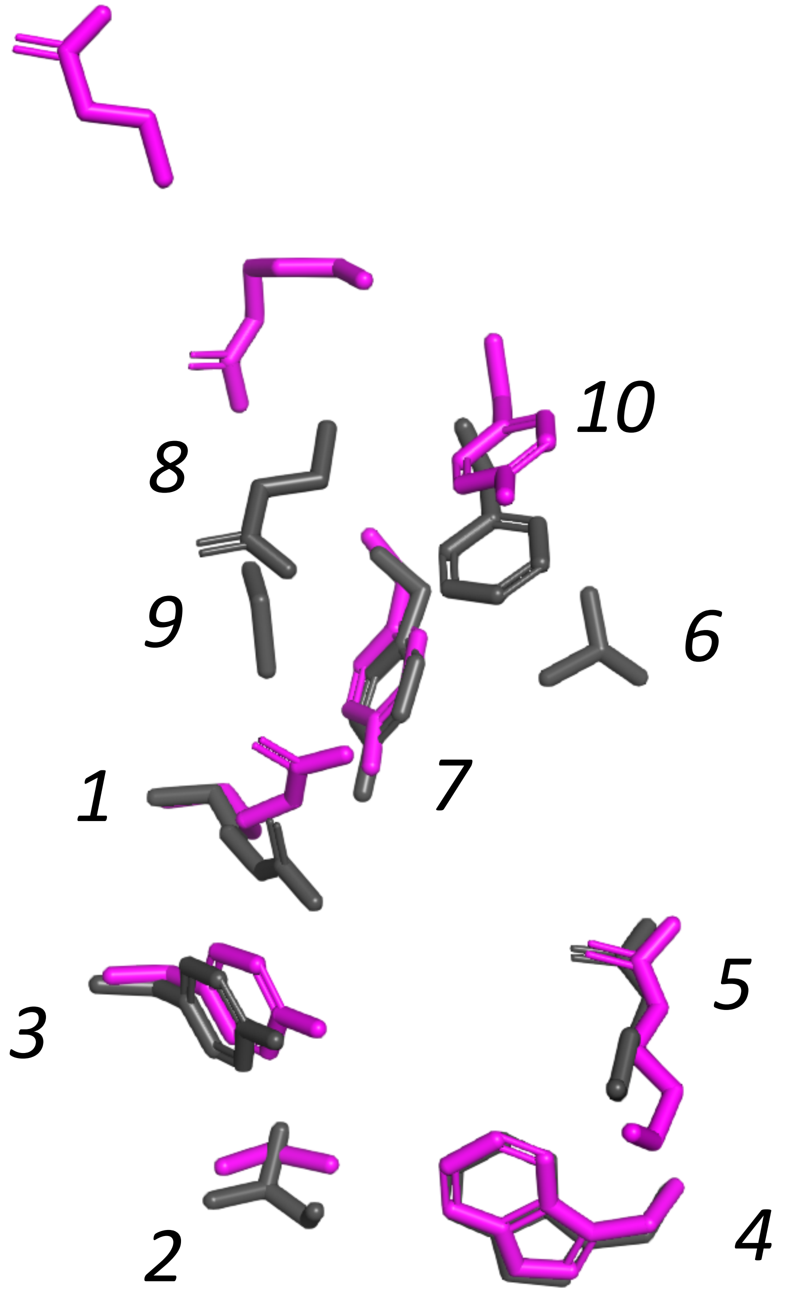
10
8
9
6
1
7
5
3
2
4

Supplemental Figure S1: A model of PP7 rationalizes insensitivity to okadaic acid.
Structural alignment of the conserved toxin binding residues of HsPP1γ and AtPP7 was produced from the solved PP1γ structure (dark gray; PDB: 1JK7) and an AlphaFold (35) generated model of Arabidopsis thaliana PP7 (magenta; At5g63870); toxin binding residues (sites1-10, see Tables 1 and 2) were extracted and visualized in PyMOL.

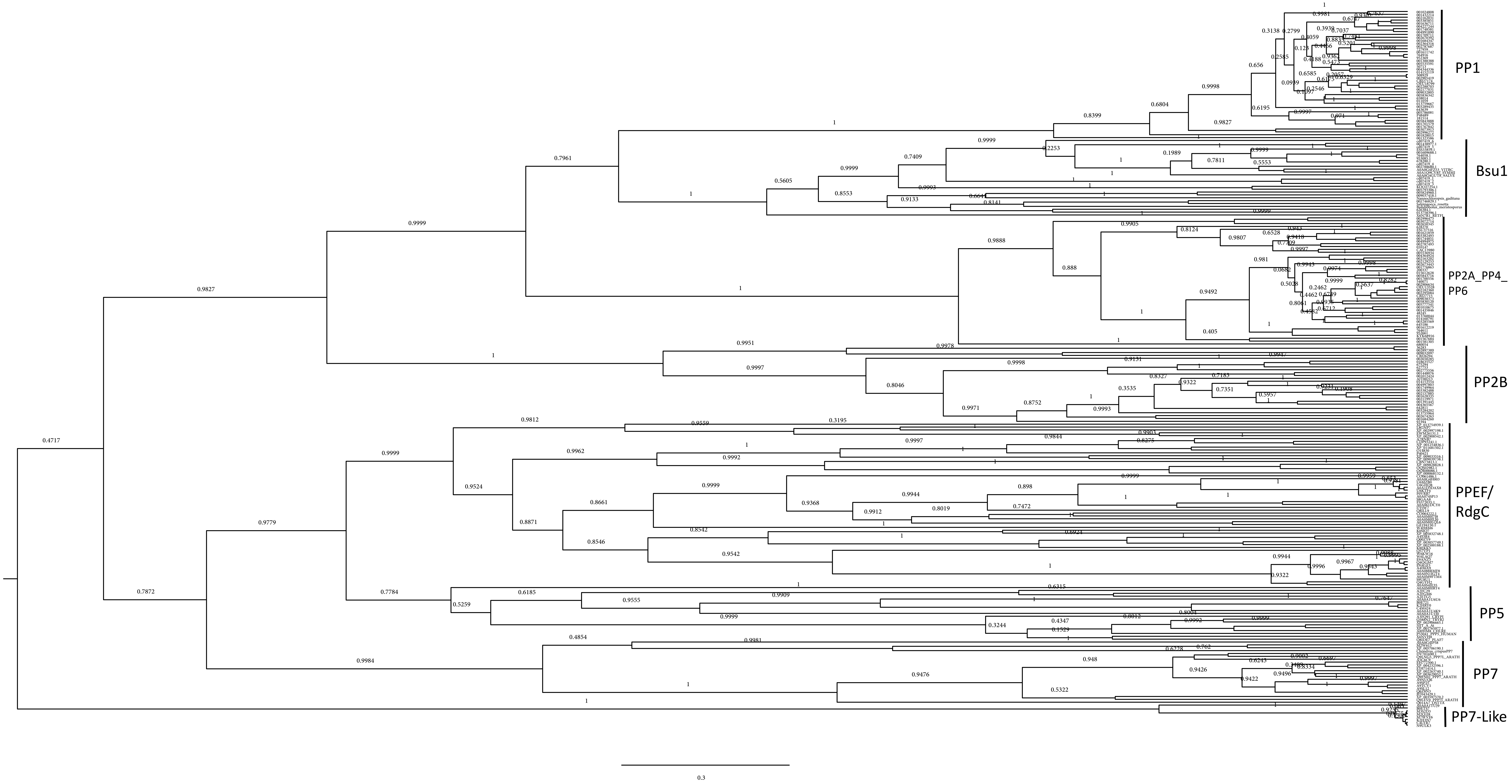
PP1
Bsu1
PP2A_PP4_
PP6
PP2B
PPEF/
RdgC
PP5
PP7
PP7-Like
0.3

Supplemental Figure S2: Detailed Toxin-Sensitivity Evolution Tree for Eukaryotic PPPs. Reference Eukaryotic PPP (phosphoprotein phosphatase) sequences were collected from the literature and from a search of a database from a panel of 45 completely sequenced Eukaryotic genomes utilizing Eukaryotic PPP HMMs (Hidden Markov Models) as detailed in Methods. Candidate novel sequences for Eukaryotic PPP subtypes were collected from an iterative database search utilizing Eukaryotic PPP HMMs. Sequences were aligned, and a rooted Bayesian tree inferred as detailed in Methods. The organisms represented by each wedge are shown. Support values are posterior probabilities. Eukaryotic PPP subtypes are indicated.
